# Are Molar-Incisor Hypomineralization and Hypomineralized Second Primary Molars Predictive of Dental Caries?: A Systematic Review

**DOI:** 10.1055/s-0042-1749360

**Published:** 2022-09-19

**Authors:** Mouna Ben Salem, Farah Chouchene, Fatma Masmoudi, Ahlem Baaziz, Fethi Maatouk, Hichem Ghedira

**Affiliations:** 1 Faculty of Dental Medicine of Monastir, Department of Pediatric and Preventive Dentistry, Monastir, Tunisia; 2 Laboratory of Biological, Clinical and Dento-Facial Approach, University of Monastir, Monastir, Tunisia

**Keywords:** dental enamel hypoplasia, tooth, deciduous, tooth demineralization, child, prevalence, dental caries

## Abstract

To estimate the prevalence of dental caries among children affected concomitantly with molar-incisor hypomineralization (MIH) and hypomineralized second primary molars (HSPM).

Electronic databases, including Medline via PubMed, Cochrane Library, Scopus, and Science Direct, were searched. Studies written in English involving children with MIH-HSPM and dental caries were considered eligible. Two reviewers extracted the data according to the PRISMA statement and assessed the bias risk with the Newcastle–Ottawa Scale (NOS) criteria.

Out of 535 studies identified in the initial research, only two articles were included in the present review. A significant association between MIH-HSPM and dental caries in permanent dentition was reported in the two included records (AOR: 3.70; *p* < 0.001). Children with MIH-HSPM expressed significantly higher DMFT and PUFA values than children without MIH-HSPM. However, dmft values did not differ significantly between children with and without HSPM (1.79 [2.4] vs. 1.78 [2.5]; 1.88 [2.73] vs. 1.59 [2.53], respectively.

MIH-HSPM lesions were correlated with a higher prevalence and more severe carious lesions in permanent dentition. However, the results should be interpreted cautiously because the findings were generated by only two studies performed by the same authors. Thus, further well-designed studies are highly recommended.

## Introduction

Molar incisor hypomineralization (MIH) is usually defined as a qualitative defect of systematic origin with hypomineralization affecting one to four first permanent molars (FPMs), commonly associated with permanent incisors. 1 Hypomineralized second primary molars (HSPM) are MIH-like defects localized in one to four-second primary molars (SPMs). 2

MIH and HSPM are considered eminent oral health conditions universally affecting children, with a prevalence rate ranging from 2.5% to 40% for MIH and from 0 to 21.8% for HSPM. 3 The diversity of study populations and the shortage of standardization in the investigation protocols can explain this heterogeneity in the findings. Results are still controversial and none of the published research has succeeded to identify clear etiological conditions for MIH and HSPM.

Environmental factors and genetic effects can concomitantly lead to MIH and HSPM occurrence. 4 FPMs and SPMs have a simultaneous period of mineralization as a result risk factors arising during this period could affect those teeth concomitantly. 5 MIH and HSPM association was widely assessed in the literature. A systematic review published in 2018 showed that HSPM was a predictive sign for MIH, with higher MIH prevalence associated with the presence of mild HSPM. 6 MIH and HSPM affected-children are susceptible to expeditious dental caries experience. The porous structure may facilitate the accumulation of bacterial plaque consequently promoting dental caries formation.

Even though the mechanism involved in the hypomineralized teeth hypersensitivity is still ambiguous, Fagrell et al (2008) have reported that the high porosity of hypomineralized teeth widely promotes the penetration of bacteria in the dentinal tubules, causing subclinical pulpal inflammation. 7 8 The discomfort reported by the patients may be spontaneous or caused by thermal or mechanical stimuli. 9 Although a few studies investigated this phenomenon, recent findings have shown that sensitivity is more frequent and intense in severe MIH with post-eruptive enamel breakdown (PEB). This finding is still controversial because of the high frequency of carious lesions involving dentine in severe hypomineralized teeth. 9 MIH-HSPM children repel tooth brushing because of the discomfort related to hypersensitivity, which can exacerbate caries vulnerability. This is in line with a previous systematic review published in 2017 that showed a high association between MIH and dental caries. 10 Recently, a significant association between HSPM and dental caries has been assessed. 11 12 Therefore, children with HSPM and MIH may be more susceptible to severe carious lesions than non-affected ones.

The present review aimed to determine the prevalence of dental caries among children affected simultaneously with MIH and HSPM.

## Methods

### Protocol and Registration

The present systematic review was designed and conducted following the Preferred Reporting Items for Systematic Reviews and Meta-Analyses (PRISMA statement) checklist 13 and was recorded in the International Prospective Register of Systematic Reviews (PROSPERO database) under the protocol CRD42020210028.

### Present Research Question

The review question was “Are children affected concomitantly with MIH and HSPM at high risk for developing dental caries?” This question was based on the Population, Intervention, Comparators, Outcomes, and Study design (PICOS) format, resulting in the following ( Table 1 ):

-P: the population was: children older than 6 years, in mixed dentition with MIH-HSPM and dental caries were included in the present review.

-I: The intervention: we searched for the type of association between Molar incisor hypomineralization, hypomineralized second primary molars, and dental caries.

C: the comparators: were the control groups of the studies.

O: The main outcome or endpoint of interest was the prevalence of carious lesions in MIH-HSPM children.

S: The study design: observational studies.

**Table 1:** The PICOS format illustrating the review question:

Population	Children older than 6 years, in mixed dentition, with MIH-HSPM and dental caries were included in the present review. Children aged under 6 years or older than 12 years were excluded.
Intervention	Type of association between molar incisor hypomineralization, hypomineralized second primary molars and dental caries. Publications that assessed only MIH and dental caries or studies interested in HSPM and dental caries without MIH assessment were excluded.
Comparators	The control groups of the studies
Outcomes	The main outcome or endpoint of interest was the prevalence of carious lesions in MIH-HSPM children.
Study design	Observational studies were included. Case reports, narrative studies (dissertations), literature reviews, meta-analyses, systematic reviews, clinical practice guidelines, theses, chapters of textbooks or textbooks, brief communications, and annals of congress were excluded.

### Inclusion and Exclusion Criteria

Inclusion and Exclusion criteria are summarized in Table 1 . Studies that pooled children affected with MIH, HSPM, and dental caries were included in the present systematic review:

-Studies that included children in mixed dentition, reported the prevalence of dental caries in children affected with both MIH and HSPM.
-Studies that assessed the outcomes of dental caries using the DMFT (decayed, missing, and filled teeth) index as described by the World Health Organization (WHO); the International Caries Detection and Assessment System (ICDAS II) or Pulp, ulceration, fistula, abscess (PUFA/pufa) index.
-Studies that evaluated the outcomes of MIH and HSPM using the European Academy of Pediatric Dentistry (EAPD) index or the Modified Index of Developmental Defects of Enamel.

The following publications were excluded:

- Publications that assessed only MIH and dental caries or studies interested in HSPM and dental caries without MIH assessment.
- Studies that involved children aged under 6 years or older than 12 years, studies that did not answer the review question, and studies written in a language other than English.
- Case reports, narrative studies (dissertations), literature reviews, meta-analyses, systematic reviews, clinical practice guidelines, theses, chapters of textbooks or textbooks, brief communications, and annals of congress.

### Information Sources and Search Strategy

Two authors (B.S.M. and C.F.) used four online databases: Medline via PubMed, Cochrane Library, Scopus, and Science Direct to identify pertinent studies. The two authors reviewed the gray literature using an advanced Google search. The first 50 PDFs derived were screened for eligibility to identify additional studies. The search was limited to studies published in English without restrictions on publication year. The initial search for articles was performed on January 20, 2021, and a subsequent search was conducted on December 01, 2021.

The reference sections of full-text records were also hand-screened by the two authors to identify further eligible publications.

A selection of search terms, Medical Subject Heading terms (MeSH), and keywords were established and adapted for each database. Only terms related to MIH, HSPM, and dental caries were used to identify eligible studies. The set of keywords used during the search is given in Table 2 .

**Table 2:** Keywords used to develop the search strategies

Database	Search strategy	Results
PubMed	-”Dental Enamel Hypoplasia”[Mesh] AND “Tooth, Deciduous”[Mesh] AND “Tooth Demineralization”[Mesh] AND “Child”[Mesh] AND “prevalence”[Mesh]. - (“Molar”[Mesh] OR “Incisor”[Mesh]) AND “Dental Enamel Hypoplasia”[Mesh] AND “Tooth Demineralization”[Mesh]) AND “Child”[Mesh] AND ”prevalence”[Mesh]. -Dental Enamel Hypoplasia”[Mesh]) AND “Tooth, Deciduous”[Mesh]) AND “Prevalence”[Mesh]) AND “Child”[Mesh] AND “prevalence”[Mesh]. - “Dental Enamel Hypoplasia”[Mesh]) AND “Tooth, Deciduous”[Mesh] AND “Tooth Demineralization”[Mesh] AND “Child”[Mesh] AND “Dental Caries”[Mesh] AND “prevalence”[Mesh]. - (“Molar”[Mesh] OR “Incisor”[Mesh]) AND “Dental Enamel Hypoplasia”[Mesh] AND “Tooth Demineralization”[Mesh]) AND “Child”[Mesh] AND “Dental Caries”[Mesh] AND “prevalence”[Mesh]. -Dental Enamel Hypoplasia”[Mesh] AND “Tooth, Deciduous”[Mesh] AND “Prevalence”[Mesh] AND “Child”[Mesh] AND “Dental Caries”[Mesh].	218
Scopus	-(“Molar incisor hypomineralization” OR “Dental enamel hypoplasia” OR “MIH”) AND (“Hypomineralized second primary molars” OR “Deciduous molar hypomineralization” OR “HSPM”) AND “prevalence.” -(“Molar incisor hypomineralization” OR “Dental enamel hypoplasia” OR “MIH”) AND (“Hypomineralized second primary molars” OR “Deciduous molar hypomineralization” OR “HSPM”) AND “Dental caries” AND “prevalence” -(“Molar incisor hypomineralization” OR “Dental enamel hypoplasia” OR “MIH”) AND “Dental caries” AND “prevalence.” -(“Hypomineralized second primary molars” OR “Deciduous molar hypomineralization” OR “HSPM”) AND “Dental caries” AND “prevalence.”	117
Science Direct	-(“Molar incisor hypomineralization” OR “Dental enamel hypoplasia” OR “MIH”) AND (“Hypomineralized second primary molars” OR “Deciduous molar hypomineralisation” OR “HSPM”) AND “prevalence.” -(“Molar incisor hypomineralization” OR “Dental enamel hypoplasia” OR “MIH”) AND (“Hypomineralized second primary molars” OR “Deciduous molar hypomineralization” OR “HSPM”) AND “Dental caries” AND “prevalence.” -(“Molar incisor hypomineralization” OR “Dental enamel hypoplasia” OR “MIH”) AND “Dental caries” AND “prevalence.” -(“Hypomineralized second primary molars” OR “Deciduous molar hypomineralization” OR “HSPM”) AND “Dental caries” AND “prevalence.”	90
Cochrane Library	#1Molar incisor hypomineralization #2dental enamel hypoplasia #3MIH #4Hypomineralised second primary molars #5Deciduous molar hypomineralization #6HSPM #7prevalence #8 #1 OR #2 OR #3 AND #4 OR #5 OR 6 AND #7 #9Dental caries #10 #8 AND #9 #11 #1 OR #2 OR #3 AND #7 AND #9 #12 #4 OR #5 OR #6 AND #7 AND #9	108

### Study Selection

Zotero 5.0.59 software was used to manage bibliographies, citations, and duplicates. Three-stage publications' selection was managed by the two authors. First, to eliminate obviously irrelevant references, only the titles of studies were considered for eligibility by the authors. Second, the authors excluded irrelevant studies based on their abstract.

The ultimate stage was based on a full-text assessment of the remaining articles from the second stage. The reference sections of the included studies assessed for eligibility were manually searched to identify supplemental records by the two authors. Disaccords were resolved by a senior author (F.M.) and a discussion between the three reviewers (M.B.S., F.C., and F.M.).

### Outcome Variables

In the present systematic review, the authors considered the prevalence of carious lesions in MIH-HSPM children as the main outcome and the severity of dental caries in those children as the secondary outcome, for a direct assessment of the caries susceptibility among MIH-HSPM affected children.

### Data Collection Process

A spreadsheet (Excel 2013, Microsoft), performed based on the Cochrane Handbook for systematic reviews checklist, was used to extract data. The data extraction was conducted independently by the two reviewers (M.B.S. and F.C.) and any disagreements were discussed until a consensus was reached.

The following items were summarized: publication details (author name, year of publication, and country), study characteristics (study design, sample size, and sample age), examination conditions, diagnostic criteria, the prevalence of MIH, HSPM, dental caries, and the association between MIH, HSPM, and dental caries.

### Quality Assessment

The quality assessment of the included studies was performed independently by the two authors using an adapted form of the Newcastle Ottawa cohort scale for cross-sectional studies. 14 This scale includes seven items with ten stars assigned over three categories: selection (maximum 5 stars), comparability (maximum 2 stars), and evaluation of outcome (maximum 3 stars).

A study was estimated as high quality if the total mark was ranging seven or higher. To assess the risk of bias, the authors determined methodological cut-off points: the primary confounder (the letter “a” under comparability) was attributed to dental caries in the primary dentition, as it has a pertinent effect on the etiology of carious lesions in the permanent teeth. Further possible confounders (letter “b” under “comparability”) were attributed to further risk factors related to caries formation in permanent teeth, for instance, socioeconomic status and age. Concerning the outcome rating (item 1 under outcome) the independent blind assessment was represented by the estimation of dental caries, MIH, and HSPM by distinct examiners. 10 It was difficult for an examiner to assess the outcome (carious lesions) without perceiving the exposures (MIH, HSPM). Finally, the statistical test (item, “2” under outcome) measured the association using confidence intervals and the probability level ( *p-* value).

## Results

### Study Selection

A total of 535 eligible articles were selected: 218 from PubMed, 108 from Cochrane Library, 117 from Scopus, 90 from Science Direct and two publications were added from a manual search. After removing 252 duplicates, titles, and abstracts of 283 studies were screened in the first phase and 236 articles were excluded based on the selection criteria. The references of the 47 remaining articles were screened to find out further records.

**Fig. 1:**
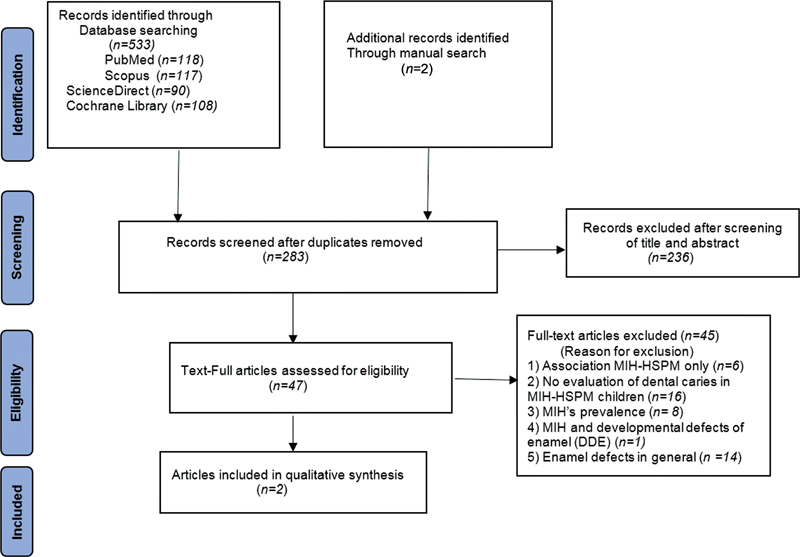
PRISMA flow diagram showing systematic review process This figure described the process of systematic search throughout different online databases to seek eligible studies. Only two articles were included finally.

After reading the full text of the selected 47 records, only two articles were included in the present review for qualitative synthesis. The PRISMA flow diagram summarizing the search method and the article selection process is shown in Fig. 1 .

### Study Characteristics

The descriptive characteristics and results of the two articles are summarized in Table 3 . The included publications were published between 2018 and 2019 and were performed in Chile 15 and Australia. 16 The two included articles were cross-sectional in design and were conducted in primary schools amongst children aged between 6 and 12 years.

**Table 3:** Characteristics of the included studies

Study	Study design	Sample characteristics	Examination Conditions	Diagnostic criteria for MIH/HSPM	Diagnostic criteria for dental caries	MIH prevalence	HSPM prevalence	MIH-HSPM association	Caries prevalence	Association MIH/ HSPM and dental caries	Main outcomes
K.Gambetta- Tessini et al. 2018/ Australia 16	Cross-sectional	327 children Males: 173 Females: 154 6–12 (years)	Two Catholic schools and Nine public schools/ Teeth dried/ External light source+ disposable examination Kits.	Modified EAPD protocol	DMFT/dmft index ICDAS II index PUFA/pufa index	14.7% (48/327)	8% (26/327)	OR: 2.90 *p* -value: 0.02	MIH/HSPM: DMFT mean (s.d.): 1.08 (1.86) dmft mean (s.d.): 1.88 (2.73) PUFA mean (s.d.): 0.02 (0.15) pufa mean (s.d.): 0.26 (0.78)	AOR: 2.17 p-value < 0.001	-MIH-HSPM children had a higher prevalence and more severe dental caries compared with non-affected ones. -Significant association between MIH and HSPM.
									MIH/HSPM free: DMFT mean (s.d.): 0.53 (1.29) dmft mean (s.d.): 1.59 (2.53) PUFA mean (s.d.): 0 pufa mean (s.d.): 0.18 (0.97)		
K.Gambetta- Tessini et al. 2019/ Chile 15	Cross-sectional	577 children Males: 285 Females: 292 6–12 (years)	Three schools: public, sub-sidised and private/ teeth brushed and dried/ Artificial light.	Modified EAPD protocol	DMFT/dmft index ICDAS II index PUFA/pufa index	15.8% (91/577)	5% (29/577)	AOR: 3.7 *p-value* : 0.001	MIH/HSPM: DMFT mean (s.d.): 1.28 (1.7) dmft mean (s.d.): 1.79 (2.4) ICDAS II > 0: 89 (6.8) PUFA mean (s.d.): 0.18 (0.5) pufa mean (s.d.): 0.24 (0.6) PUFA + pufa prevalence: 24.1%	AOR: 3.70 *p* -value < 0.001	-Positive association between: -MIH-HSPM, and dental caries prevalence and severity. -MIH and HSPM.
									MIH/HSPM free: DMFT mean (s.d.): 0.76 (1.4) dmft mean (s.d.): 1.78 (2.5) ICDAS II > 0: 1222 (93.2) PUFA mean (s.d.): 0.27 (0.8) pufa mean (s.d.): 0.27 (0.8)		

Abbreviations: AOR, adjusted odds ratio; DHL, demarcated hypomineralized lesions; dmft, decayed missing due to caries and filled teeth in primary teeth; DMFT, decayed missing due to caries and filled teeth in the permanent teeth; HSPM, hypomineralized second primary molars; ICDAS II, international caries detection and assessment system II; MIH, molar incisor hypomineralization; OR, odds ratio; P, *p* -Value; PUFA, pulp ulceration fistula abscess teeth in primary teeth; SD/sd, standard deviation

### Assessment of Risk of Bias

Using the adapted form of the Newcastle Ottawa cohort scale for cross-sectional studies, the two included studies were rated as high-quality studies ( Table 4 ). The record conducted by Gambetta-Tessini et al in 2019 scored 9 stars and the study conducted by the same authors in 2019 had a score of 7 stars. 15 16

**Table 4:** Quality assessment using the Newcastle-Ottawa Scale adapted for cross-sectional studies

Author Year Country	Study Design	Selection 1 2 3 4	Comparability 5a 5b	Outcomes 6 7	Total
Gambetta-Tessini et al 2018/ Australia 16	Cross-sectional Study	* * * **	* *	* *	9 High quality
Gambetta-Tessini et al 2019/ Chile 15	Cross-sectional Study	* **	* *	* *	7 High quality

Note: Selection 1: Representativeness of the sample; Selection 2: Sample size; Selection 4: Ascertainment of the exposure; comparability: Comparability of subjects in different outcome groups based on the study design or analysis. Confounding factors are controlled; Outcome 1: Assessment of the outcome; Outcome 2: Statistical test

### Main Outcomes

MIH and HSPM outcomes were assessed by the two authors using the modified EAPD protocol. The outcomes of MIH and HSPM were represented by prevalence and percentages.

Carious lesions were recorded according to the criterion settled by the WHO, 17 the ICDAS II scoring criteria, 18 and PUFA/pufa. 16

Regarding the association between MIH, HSPM, and its impact on dental caries, statistical tests were used represented by *p-value* and adjusted odds ratio (AOR) in the included records ( Table 3 ).

As shown in Table 3 , the two pooled articles found a positive association between MIH/HSPM and dental caries. 15 16

It was principally represented by a high AOR: 3.70 and *p* < 0.001. 15 16

The authors found a higher prevalence of dental caries in MIH/HSPM children compared with non-affected ones. This was represented by a higher DMFT mean (SD) expressed by those affected children compared with those who were not affected, and the values ranged from 1.08 (1.86) to 1.28 (1.7). 15 16

The dmft mean (SD) ranged from 1.79 (2.4) to 1.88 (2.73) in demarcated hypomineralized lesions-affected children. 15 16 However, the authors stated that dmft did not differ significantly between those infants with and without hypomineralized primary teeth. 15 16

PUFA mean values were 0.02 (s.d. = 0.15) 16 and 0.18 (s.d. = 0.5) 15 among affected children compared with 0 16 and 0.01 (s.d. = 0.1) 15 among non-affected children.

Concerning pufa mean values, Gambetta-Tessini et al in 2019 found similar results in both hypomineralized and defect-free teeth, values were 0.24 (s.d. = 0.6) and 0.27 (s.d. = 0.8) respectively. In the second study, pufa were slightly higher in MIH-HSPM affected group compared with non-affected ones, values were 0.26 (s.d. = 0.78) and 0.18 (s.d. = 0.97) respectively.

The prevalence of hypomineralized teeth with severe carious lesions was 14.6% compared with 2.2% of defect-free teeth. 15 The second study 16 reported that 9.3% of demarcated hypomineralized lesions were diagnosed with severe carious lesions compared with 1.9% of unaffected teeth.

Therefore, the two publications showed that MIH-HSPM defects increased the severity of the carious lesions as a high OR (2.17) and a significant *p* -value (< 0.001) were reported. 15 16

## Discussion

The present review aimed to determine the effect of MIH/HSPM lesions on the dental caries experience. MIH may be considered one of the risk factors for dental caries. Therefore, children with hypomineralized teeth may need regular monitoring to prevent dental caries extension.

The sample size of the included studies ranged from 327 to 577 children, which was in line with the literature that recommended a standardized evaluation of MIH/HSPM prevalence using a sufficiently explicit sample involving a minimum of 300 candidates. 19

The age of candidates in this review ranged from 6 to 12 years. It has been recommended to evaluate MIH at 8 years of age when all molars and incisors have barely erupted 10 restricting the risk of enamel defects concealment by carious cavities or restorations.

For HSPM, the age of 5 years could be convenient for detecting enamel defects because most SPMs fully erupt at this stage and HSPM are distinctly perceptible. Also, at this age children are relatively more cooperative allowing proper oral examinations. The non-difference in dmft values between children with or without hypomineralized teeth and the similar pufa values in the study of Gambetta Tessini et al in 2018 can be related to the advanced sample age. 15 16 Currently, between 6 and 12 years, SPMs are highly exposed to prolonged environmental risk factors exacerbating the defect severity into PEB and carious lesions. The probability of confusing PEB arising from hypomineralization as the main cause in teeth with severe dental caries exists due to the relatively advanced age. This may underestimate the true number of teeth with PEB because of hypomineralization as a primary outcome. Also, restorations may conceal primary hypomineralization and even the common absence of those teeth at this age group may explain this HSPM underestimation. Owen et al find similar results and reported that HSPM presence did not predispose those children to greater caries risk; the authors explained their findings by the overall low caries prevalence in their study resulting in a limited number of HSPM with cavitated lesions. 20 Thus, the current results reporting HSPM prevalence and its association with dental caries should be interpreted cautiously. However, many authors reported a positive association between HSPM and dental caries among samples of similar age, e.g., 7-to-9-year-old children. 11 12

In the present review, the two included studies 15 16 showed that children with MIH and HSPM express a higher dental caries prevalence in permanent dentition. This was mainly represented by a higher DMFT index and a significant *p* -value less than 0.05. It is well known that porosity and poor mechanical resistance have been increased by enamel hypomineralization, especially in the case of PEB. 21 Thus, hypomineralized lesions facilitate bacterial plaque agglomeration and increase dental cavities development.

Teeth with severe hypomineralized defects present, hypersensitivity that results in tooth brushing avoidance. Thus, children are more susceptible to dental caries, especially in cases of poor oral health, high sugar consumption, and low salivary buffer capacity. 1 15 21

In addition, the association between MIH, HSPM and dental caries can be explained by common etiological factors. In fact, Vieira and Kup. (2016) in their systematic review have demonstrated that a genetic mutation may occur during dental enamel formation, causing disturbances in the maturation stages of enamel. 22 These disturbances mostly affect the first permanent molars and incisors and may consequently lead to the MIH occurrence. SPMs, permanent canines, and premolars may also be involved. Other genetic variations, in any of the above 100 genes expressed over late enamel formation, can explain the involvement of further teeth.

Genetic factors contribute likely to dental caries etiology. 23 Mutations in genes such as dentine sialophosphoprotein mutation lead to abnormal proteins or decrease the load of these proteins in immature teeth. Therefore, inadequate mineralization arises and possibly affects both bacterial adherence and resistance of enamel to an acid pH; so, the vulnerability of tooth surfaces to carious lesions is increased. 23

Some authors have suggested that mutations in kallikrein-related peptidase 4 (KLK4) gene expression is implicated in more porous and weak enamel formation, leading to hypomineralization and caries. This can be explained by the fact that KLK4 secretes enamel matrix protein. 22 23 Variations in genes, which produce enamel mineralization, may increase the susceptibility to MIH, HSPM, and dental caries simultaneously. However, environmental factors play a significant role in the etiology of dental caries (e.g., sugar consumption, poor oral health, oral bacteria and malocclusions), 24 25 MIH and HSPM (e.g., health during pregnancy, delivery complications, birth weight, childhood illness). 26 27 Therefore, additional studies in various populations are required to determine the part of both environmental and genetic factors in those dental issues. Thus, susceptible patients could be identified earlier in the future. 28

The present review demonstrated also that not only the caries prevalence but also the severity of the carious lesions can be significantly associated with the enamel hypomineralization. A high odds ratio was reported in the study conducted by Gambetta-Tessini et al in 2019 and the prevalence of MIH/HSPM affected teeth with severe carious lesions was 14.6% compared with 2.2% of the non-affected ones. 15 This may be related to the rapid carious lesions progress in the case of hypomineralized enamel because of its overly altered and delicate structure. However, other factors like poor oral hygiene and access constraints to oral care can be implicated in caries severity. 16 In addition, a higher PUFA experience was reported in MIH-HSPM affected groups. Children affected with hypomineralized lesions such as MIH and HSPM present in most cases pulp involvement requiring endodontic treatment. 29 30

The two included studies 15 16 reported that hypomineralized lesions were correlated with a higher dental caries severity, especially amongst children from low socioeconomic status. This was in line with recent publications which illustrated a significant association between MIH, dental caries risk, and socioeconomic factors. 31 In fact, families from low SES express unfavorable oral hygiene and restricted access to both preventive measures and restorative treatment. Leading caries to progress more rapidly into severe lesions. Therefore, health authorities should provide and facilitate dental services access to disadvantaged families through preventive oral health companies and programs.

In addition, the two pooled publications reported that HSPM represents a predictive sign for MIH. This was mainly illustrated by high odds ratios and significant *p* -value. Those findings agreed with a previous systematic review and meta-analysis. 6 Actually, the crown calcification of SPMs begins on the 18th week *in utero* and lasts for 1 year after childbirth, while for the first permanent molars the crown mineralization starts at the end of the third gestational trimester and persists for three years after childbirth. 32

Therefore, the second deciduous molars and the first permanent molars can be concomitantly affected by the risk factors acting during the simultaneous terms of mineralization. As a result, primary and permanent dentition may undergo hypomineralization defects. 5

Regarding the quality assessment, an adapted form of the Newcastle Ottawa Scale for cross-sectional studies was used as all records included in this review are cross-sectional in design. The authors predetermined methodologically appropriate cut-off points for the evaluation. To eliminate the probability of subjectiveness, the scores were granted when there was adequate information, and the reviewers were familiarized with the NOS.

Commonly, assessments are esteemed blinded if the examiner assesses outcomes without realizing the presence or not of the exposure. In the present studies, it was difficult for the examiner to evaluate caries (outcome) without seeing MIH (exposure). Therefore, we reviewed blind assessment as performed when MIH and dental caries were evaluated by two distinct examiners. 10 None of the studies followed this condition; consequently, the risk of bias can arise restricting the validity of the current surveys.

Potential confounders such as age, gender, region, sample size, and socioeconomic status were considered in the two studies. This may also occasion biased findings. 15 16

Although all pooled studies were recorded as high quality using the NOS scale, we can identify some limitations related to those surveys, e.g., differences in diagnostic criteria compared with other studies, especially, that the index which was used by the authors is recently employed in the literature. Although it has been known as a valid diagnostic index, 33 Lopes et al have reported that the use of alternative classification, other than the EAPD criterion, generates a reduction in MIH prevalence. 34 Thus, a standardization of the employed index is recommended to reduce heterogeneity in MIH/HSPM findings. 34 As explained earlier outcomes about HSPM prevalence and association with dental caries need cautious interpretation as in literature the optimal age for HSPM assessment is 5 years.

Concerning the current systematic review, we cannot neglect some restrictions, e.g., the same authors conducted the two included studies. This did not influence their characteristics as they had been conducted separately and displayed different populations, countries, and outcomes. However, biased results may occur. Also, few studies have been interested in the effect of MIH and HSPM on carious lesions experience, so the results need cautious interpretation. Thus, pertinently future studies are particularly necessary to issue further confirmation about this topic.

## Conclusion and Recommendations

To the best of our knowledge, the present systematic review is the first report aiming to assess the caries experience among MIH-HSPM affected children. Those infants present a higher prevalence and more severe carious lesions in permanent dentition.

Although in this review, dental caries experience does not differ between children with or without HSPM, clinicians should give more intention and regular follow-up at more frequent intervals for children with hypomineralized primary teeth as they may have a risk to develop dental caries, poor oral hygiene, and MIH in permanent dentition. Therefore, prevention should be performed directly after hypomineralized teeth eruption. Dietary advice, fluoride toothpaste with a minimum of 1450 ppm (ppm), and professional application of resin-based fissure sealants for intact hypomineralized molars should be programmed for MIH affected children to minimize caries risk and tooth hypersensitivity. 35

Dentists must receive ongoing training on this topical issue. In addition, there is a need to perform well-designed studies in different populations to assess the part of both environmental and genetic factors in the etiology of MIH, HSPM, and dental caries. As follows, susceptible patients can be identified earlier in the future.

## References

[1] Weerheijm K L Jälevik B Alaluusua S Molar-incisor hypomineralisation Caries Res 2001 35 05 390 391 11641576 10.1159/000047479

[2] Elfrink M EC Schuller A A Weerheijm K L Veerkamp J SJ Hypomineralized second primary molars: prevalence data in Dutch 5-year-olds Caries Res 2008 42 04 282 285 18523388 10.1159/000135674

[3] da Silva Figueiredo Sé M J Ribeiro A PD Dos Santos-Pinto L AM de Cassia Loiola Cordeiro R Cabral R N Leal S C Are hypomineralized primary molars and canines associated with molar-incisor hypomineralization? Pediatr Dent 2017 39 07 445 449 29335050

[4] Silva M J Scurrah K J Craig J M Manton D J Kilpatrick N Etiology of molar incisor hypomineralization - a systematic review Community Dent Oral Epidemiol 2016 44 04 342 353 27121068 10.1111/cdoe.12229

[5] Temilola O D Folayan M O Oyedele T The prevalence and pattern of deciduous molar hypomineralization and molar-incisor hypomineralization in children from a suburban population in Nigeria BMC Oral Health 2015 15 73 26121979 10.1186/s12903-015-0059-x PMC4486434

[6] Garot E Denis A Delbos Y Manton D Silva M Rouas P Are hypomineralised lesions on second primary molars (HSPM) a predictive sign of molar incisor hypomineralisation (MIH)? A systematic review and a meta-analysis J Dent 2018 72 8 13 29550493 10.1016/j.jdent.2018.03.005

[7] Tonguc M O Ozat Y Sert T Sonmez Y Kirzioglu F Y Tooth sensitivity in fluorotic teeth Eur J Dent 2011 5 03 273 280 21769268 PMC3137440

[8] Fagrell T G Lingström P Olsson S Steiniger F Norén J G Bacterial invasion of dentinal tubules beneath apparently intact but hypomineralized enamel in molar teeth with molar incisor hypomineralization Int J Paediatr Dent 2008 18 05 333 340 18328044 10.1111/j.1365-263X.2007.00908.x

[9] Raposo F de Carvalho Rodrigues A C Lia ÉN Leal S C Prevalence of hypersensitivity in teeth affected by molar-incisor hypomineralization (MIH) Caries Res 2019 53 04 424 430 30677762 10.1159/000495848

[10] Americano G CA Jacobsen P E Soviero V M Haubek D A systematic review on the association between molar incisor hypomineralization and dental caries Int J Paediatr Dent 2017 27 01 11 21 27098755 10.1111/ipd.12233

[11] Ghanim A Manton D Mariño R Morgan M Bailey D Prevalence of demarcated hypomineralisation defects in second primary molars in Iraqi children Int J Paediatr Dent 2013 23 01 48 55 22276809 10.1111/j.1365-263X.2012.01223.x

[12] Oyedele A T Folayan O M Oziegbe O E Hypomineralised second primary molars: prevalence, pattern and associated co morbidities in 8- to 10-year-old children in Ile-Ife, Nigeria BMC Oral Health 2016 16 65 10.1186/s12903-016-0225-9 PMC4893208 27259516

[13] PRISMA Group Moher D Liberati A Tetzlaff J Altman D G Preferred reporting items for systematic reviews and meta-analyses: the PRISMA statement PLoS Med 2009 6 07 e1000097 19621072 10.1371/journal.pmed.1000097 PMC2707599

[14] Herzog R Álvarez-Pasquin M J Díaz C Del Barrio J L Estrada J M Gil Á Are healthcare workers' intentions to vaccinate related to their knowledge, beliefs and attitudes? A systematic review BMC Public Health 2013 13 154 23421987 10.1186/1471-2458-13-154 PMC3602084

[15] Gambetta-Tessini K Mariño R Ghanim A Calache H Manton D J The impact of MIH/HSPM on the carious lesion severity of schoolchildren from Talca, Chile Eur Arch Paediatr Dent 2019 20 05 417 423 30637683 10.1007/s40368-019-00416-w

[16] Gambetta-Tessini K Mariño R Ghanim A Calache H Manton D J Carious lesion severity and demarcated hypomineralized lesions of tooth enamel in schoolchildren from Melbourne, Australia Aust Dent J 2018 63 03 365 373 10.1111/adj.12626 29876927

[17] Organization W H Oral health surveys: basic methods World Health Organization 1997Accessed July 21, 2020 at:https://apps.who.int/iris/handle/10665/41905

[18] Gugnani N Pandit I K Srivastava N Gupta M Sharma M International caries detection and assessment system (ICDAS): a new concept Int J Clin Pediatr Dent 2011 4 02 93 100 27672245 10.5005/jp-journals-10005-1089 PMC5030492

[19] Elfrink M EC Ghanim A Manton D J Weerheijm K L Standardised studies on molar incisor hypomineralisation (MIH) and hypomineralised second primary molars (HSPM): a need Eur Arch Paediatr Dent 2015 16 03 247 255 25894247 10.1007/s40368-015-0179-7

[20] Owen L M Ghanim A Elsby D Manton J J Hypomineralized second primary molars: prevalence, defect characteristics and relationship with dental caries in Melbourne preschool children Aust Dent J 2018 63 01 72 80 28881480 10.1111/adj.12567

[21] Ghanim A Mariño R Morgan M Bailey D Manton D An in vivo investigation of salivary properties, enamel hypomineralisation, and carious lesion severity in a group of Iraqi schoolchildren Int J Paediatr Dent 2013 23 01 2 12 10.1111/j.1365-263X.2011.01215.x 22251406

[22] Vieira R A Kup E On the Etiology of Molar-Incisor Hypomineralization Caries Res 2016 50 02 166 169 27111773 10.1159/000445128

[23] Opal S Garg S Jain J Walia I Genetic factors affecting dental caries risk Australian Dental Journal 2015 60 01 2 11 10.1111/adj.12262 25721273

[24] Sá-Pinto C A Rego M T Marques S L Martins C C Ramos-Jorge L M Ramos-Jorge J Association between malocclusion and dental caries in adolescents: a systematic review and meta-analysis Eur Arch Paediatr Dent 2018 19 02 73 82 29594971 10.1007/s40368-018-0333-0

[25] Gudkina J Amaechi T B Abrams H S Brinkmane A Jelisejeva I Caries Increment and Oral Hygiene Changes in 6– and 12–Year–Old Children in Riga, Latvia: A 3–Year Follow–Up Report Using ICDAS II and RADKE Criteria Eur J Dent 2019 13 03 413 419 31795005 10.1055/s-0039-1700250 PMC6890505

[26] Mejía D J Restrepo M González S Álvarez G L Santos-Pinto L Escobar A Molar Incisor Hypomineralization in Colombia: Prevalence, Severity and Associated Risk Factors J Clin Pediatr Dent 2019 43 03 185 189 30964726 10.17796/1053-4625-43.3.7

[27] Ghanim M A Morgan V M Mariño J R Bailey L D Manton J D Risk factors of hypomineralised second primary molars in a group of Iraqi schoolchildren Eur Arch Paediatr Dent 2012 13 03 111 118 22652207 10.1007/BF03262856

[28] emhj. Prevalence of dental caries among children aged 5–15 years from 9 countries in the Eastern Mediterranean Region: a meta–analysis. World Health Organization – Regional Office for the Eastern MediterraneanAccessed January 25, 2022 at:http://www.emro.who.int/emhj-volume-26-2020/volume-26-issue-6/prevalence-of-dental-caries-among-children-aged-515-years-from-9-countries-in-the-eastern-mediterranean-region-a-meta-analysis.html 10.6719/emhj.20.050 32621509

[29] Muratbegovic A Markovic N Ganibegovic M Selimovic Molar incisor hypomineralisation in Bosnia and Herzegovina: aetiology and clinical consequences in medium caries activity population. European archives of paediatric dentistryofficial journal of the European Academy of Paediatric Dentistry 2007 8 04 189 194 10.1007/BF03262595 18076849

[30] Bhaskar A S Hegde S Molar–incisor hypomineralization: prevalence, severity and clinical characteristics in 8– to 13–year–old children of Udaipur, India Journal of the Indian Society of Pedodontics and Preventive Dentistry 2014 32 04 322 329 25231041 10.4103/0970-4388.140960

[31] Wuollet E Laisi S Alaluusua S Waltimo-Sirén J The Association between Molar–Incisor Hypomineralization and Dental Caries with Socioeconomic Status as an Explanatory Variable in a Group of Finnish ChildrenInt J Environ Res Public Health 2018;15(07):10.3390/ijerph15071324 PMC6068618 29941779

[32] Elfrink C ME Moll A H Kiefte-de JC Jong Is maternal use of medicines during pregnancy associated with deciduous molar hypomineralisation in the offspring? A prospective, population–based study Drug Saf 2013 36 08 627 633 10.1007/s40264-013-0078-y 23743695

[33] Ghanim A Elfrink M Weerheijm K Mariño R Manton D A practical method for use in epidemiological studies on enamel hypomineralisation Eur Arch Paediatr Dent 2015 16 03 235 246 10.1007/s40368-015-0178-8 25916282 PMC4469791

[34] Lopes L B Machado V Mascarenhas P Mendes J J Botelho J The prevalence of molar-incisor hypomineralization: a systematic review and meta-analysis Sci Rep 2021 11 01 22405 10.1038/s41598-021-01541-7 34789780 PMC8599453

[35] Ghanim A Silva J M Elfrink C ME Molar incisor hypomineralisation (MIH) training manual for clinical field surveys and practice Eur Arch Paediatr Dent 2017 18 04 225 242 10.1007/s40368-017-0293-9 28721667

